# Dr. Wm. A. Keegan

**Published:** 1917-09

**Authors:** 


### OBITUARY

**Readers are requested to report promptly the death of all physicians in Western New York, or former residents of this region, or graduates of any medical school in Western New York, and to notify the families of the deceased of our desire to publish adequate Obituary notices.**

---

**Dr. Wm. A. Keegan,** Chicago Homoeopathic, 1888, died suddenly at his home in Rochester, Aug. 8, aged 56. He was born in London, came to Canada with his parents in his childhood, and was educated at the Peterboro Collegiate Institute. After graduating in medicine, he took post graduate work in Vienna and located in Rochester in 1893.

